# Development and validation of a nomogram to predict symptomatic recurrence following laparoscopic adenomyomectomy

**DOI:** 10.3389/fmed.2026.1869800

**Published:** 2026-07-16

**Authors:** Yiwen Yao, Jilan Jiang, Jin Yu, Yeping Yang, Wenyu Li, Feng Sun

**Affiliations:** 1 Department of Gynecology & Obstetrics, The International Peace Maternity and Child Health Hospital, School of Medicine, Shanghai Jiao Tong University, Shanghai, China; 2 Shanghai Key Laboratory of Embryo Original Diseases, Shanghai, China

**Keywords:** adenomyosis, laparoscopic adenomyomectomy, symptomatic recurrence, nomogram, risk prediction

## Abstract

**Introduction:**

Nomograms are intuitive graphical tools that integrate multiple prognostic variables to generate individualized risk estimates. To date, no study has developed a validated prediction model specifically for symptomatic recurrence after uterine-sparing surgery for adenomyosis. Accordingly, this study aimed to construct and validate a nomogram based on retrospective clinical data to predict symptomatic recurrence after laparoscopic adenomyomectomy and to inform individualized postoperative management.

**Materials and Methods:**

In this retrospective cohort study, 484 consecutive patients who underwent primary laparoscopic adenomyomectomy between December 1, 2017, and March 30, 2022, were included. All patients completed postoperative follow-up. Symptomatic recurrence occurred in 131 patients, while 353 remained recurrence-free. Independent predictors of recurrence were identified using multivariate Cox regression, and a nomogram was constructed. Model discrimination was evaluated using Harrell’s concordance index (C-index) and internally validated using 5-fold cross-validation. Model performance was assessed via receiver operating characteristic curve, calibration curves, and decision curve analysis. A sensitivity analysis using inverse probability of treatment weighting (IPTW) confirmed the robustness of the identified predictors against residual confounding.

**Results:**

Previous surgical history of ovarian endometrioma, preoperative CA125 level, concomitant ovarian endometrioma, postoperative medication modality, and duration of postoperative therapy were independent predictors of symptomatic recurrence. The nomogram demonstrated good discriminatory ability (area under the receiver operating characteristic curve [AUC], 0.776; 95% confidence interval [CI], 0.728–0.824). The calibration curve also had a good performance, and the DCA indicated that patients achieved a high net benefit for the predicted probability thresholds between 0% and 60%.

**Conclusion:**

This nomogram provides accurate individualized risk estimation for symptomatic recurrence after laparoscopic adenomyomectomy, which requires multicenter external validation to confirm its clinical utility.

## 1 Introduction

Adenomyosis is a common gynecologic disorder that primarily affects women of reproductive age (1). It is characterized by the presence of endometrial glands and stroma within the myometrium and commonly manifests as pelvic pain, heavy menstrual bleeding, uterine enlargement, and infertility (2).

Current management strategies include medical therapy, such as nonsteroidal anti-inflammatory drugs, progestogens, oral contraceptives, and gonadotropin-releasing hormone agonists (GnRH-a), as well as hysterectomy. Although medical therapy may alleviate symptoms, its benefits are often temporary, with frequent recurrence after discontinuation and potential adverse effects. Hysterectomy remains definitive but is unsuitable for women who desire uterine preservation or future fertility.

Uterine-sparing surgery (adenomyomectomy) offers an alternative for selected patients; however, its effectiveness in symptom control varies (3). The procedure is technically demanding due to the indistinct boundary between normal myometrium and adenomyotic tissue (4), which complicates complete lesion excision. Consequently, recurrence rates remain substantial, reaching up to 49.0% by the second postoperative year (5). Adjuvant postoperative hormonal suppression—including GnRH-a, oral contraceptives, the levonorgestrel-releasing intrauterine system (LNG-IUS), and dienogest—has been reported to reduce recurrence and improve symptom control (6).

In our previous study (7), concomitant ovarian endometrioma (OMA) was identified as a significant risk factor for symptomatic recurrence following laparoscopic adenomyomectomy, whereas postoperative hormonal suppression and age ≥ 40 years at surgery were protective factors. Despite these findings, reliable tools for individualized risk prediction remain limited. Establishing a robust predictive model to identify high-risk patients is therefore essential to guide tailored postoperative management and reduce recurrence.

Nomograms are intuitive graphical tools that integrate multiple prognostic variables to generate individualized risk estimates and have been increasingly applied in predicting disease occurrence, progression, and prognosis (8). To date, no study has developed a validated prediction model specifically for symptomatic recurrence after uterine-sparing surgery for adenomyosis. Accordingly, this study aimed to construct and validate a nomogram based on retrospective clinical data to predict symptomatic recurrence after laparoscopic adenomyomectomy and to support evidence-based clinical decision-making.

## 2 Methods

### 2.1 Study population

We retrospectively reviewed patients who underwent laparoscopic adenomyomectomy at the Department of Gynecology, International Peace Maternity and Child Health Hospital of the China Welfare Institute, between December 1, 2017, and March 30, 2022. Medical records and follow-up data were systematically collected and analyzed. We adhere to the Transparent Reporting of a Multivariable Prediction Model for Individual Prognosis or Diagnosis (TRIPOD) statement (9). The study was approved by the Institutional Review Board of the International Peace Maternity and Child Health Hospital of the China Welfare Institute (GKLW2017-71).

Eligible patients met the following criteria: (i) age 20–45 years; (ii) presence of dysmenorrhea and/or menorrhagia; (iii) laparoscopic adenomyomectomy as the primary surgical intervention; (iv) available preoperative ultrasonographic evaluation; and (v) postoperative histopathological confirmation of adenomyosis. Exclusion criteria were: (i) submucosal myoma; (ii) coexisting premalignant or malignant disease; (iii) prior surgery for adenomyosis; (iv) incomplete clinical data or refusal to participate; and (v) bilateral oophorectomy or hysterectomy.

Ultrasonographic diagnosis of adenomyosis was established according to the Morphological Uterine Sonographic Assessment (MUSA) group criteria (10). Diffuse adenomyosis was defined by widespread ectopic endometrial tissue within the myometrium, whereas focal adenomyosis was characterized by a localized hypertrophic and distorted endometrial–myometrial lesion. Ultrasonographic examinations were performed by two certified sonographers.

Dysmenorrhea severity was assessed using a visual analog scale (VAS; 0, no pain; 1–3, mild; 4–6, moderate; 7–10, severe) (11). Menstrual blood loss was evaluated using the Mansfield–Voda–Jorgensen (MVJ) menstrual bleeding scale (range 1–6), with scores ≥5 indicating menorrhagia (12). Uterine volume was calculated by transvaginal or transabdominal ultrasonography using the formula: volume = 0.5233 × D1 × D2 × D3, where D1 represents the longitudinal diameter, D2 the anteroposterior diameter, and D3 the transverse diameter. Lesion size was defined as the maximum diameter of the focal lesion on ultrasonography. Serum CA125 levels were measured by sandwich enzyme-linked immunosorbent assay (R&D Systems, Minneapolis, MN, USA) according to the manufacturer’s protocol.

All procedures were performed under general anesthesia. For focal adenomyosis, the surgical technique was similar to that used for laparoscopic myomectomy (13). In diffuse cases, the double-flap laparoscopic adenomyomectomy technique was applied (14). When coexisting endometriosis was identified, ovarian endometriomas were carefully excised, and confirmed via postoperative histopathology. Concomitant deep endometriosis (DE) or leiomyoma was also surgically removed and pathologically verified.

Given the absence of consensus regarding optimal long-term postoperative therapy to prevent recurrence, patients were counseled on available options and selected treatment according to preference. Postoperative strategies included: observation without medical therapy; levonorgestrel-releasing intrauterine system (LNG-IUS) placement (either intraoperatively or delayed by 3–6 months) (15); or other hormonal suppression, including GnRH-a for 3–6 months followed by combined oral contraceptives or dienogest. Adherence to assigned postoperative hormonal interventions throughtout the follow-up period was evaluated by retrospectively abstracting data from serial outpatient medical records. Patients were regarded as treatment-adherent when consistent clinic documentation verified sustained use of their prescribed hormonal agents as planned. Individuals with formally noted treatment pauses, premature discontinuation of hormonal medications, or inadequate medication-related documentation were categorized as non-adherent.

### 2.2 Clinical follow-up

Patients were followed either through outpatient visits or structured telephone interviews. Initial postoperative symptom improvement was assessed at least 6 months after surgery. Follow-up continued for up to 3 years postoperatively or until menopause, with serial evaluation of dysmenorrhea and menstrual bleeding patterns.

Symptom improvement was defined as a reduction in VAS score or complete resolution of pain, accompanied by normalization of menstrual volume. Symptom persistence was defined as no reduction in VAS and MVJ scores. Dysmenorrhea recurrence was defined as reappearance of pain after initial improvement, with VAS progressively ≥ 3 at least for 3 months. Menorrhagia recurrence was defined as an MVJ score ≥ 5 following a postoperative period of normalized menstruation (7).

### 2.3 Data collection

The following variables were extracted for each patient: age, body mass index, age at menarche, number of abortions, parity, delivery mode, preoperative uterine volume, lesion location,preoperative CA125, preoperative hemoglobin, preoperative VAS and MVJ scores, lesion size, multiparity status, history of abdominal surgery, history of ovarian endometrioma (OMA) surgery, failure of preoperative medical therapy, preoperative GnRH-a use, lesion type (diffuse or focal), concomitant OMA, concomitant deep endometriosis (DE), operative time, intraoperative blood loss, postoperative medication duration, postoperative hemoglobin, postoperative medication modality, duration of postoperative therapy and symptomatic recurrence.

### 2.4 Statistical analyses

All analyses were conducted using SPSS version 26.0 and R software (version 4.4.2), incorporating survival, rms, pROC, WeightIt, cobalt, caret, car, tableone, survminer, readxl, tidyverse, broom, and patchwork packages.

Continuous variables were presented as mean ± standard deviation (SD), and categorical variables as frequencies and percentages. Between-group comparisons were performed using the independent samples *t*-test for continuous variables and the chi-square or Fisher’s exact test for categorical variables.

To facilitate CA125 incorporation into the nomogram as a binary predictor, the optimal cutoff value was determined by maximizing the Youden index. Specifically, an ROC curve was constructed for continuous CA125 levels and the Youden index (sensitivity + specificity − 1) was calculated across all candidate thresholds.

Potential prognostic variables (*p* < 0.1) were identified using univariate and multivariate Cox proportional hazard analyses. Inverse probability of treatment weighting (IPTW) was applied to balance baseline characteristics. Collinearity among predictors was assessed using the generalized variance inflation factor (GVIF).

A nomogram was constructed based on the independent predictors. Model discrimination was evaluated using Harrell’s concordance index (C-index) and internally validated using 5-fold cross-validation. The performance of the nomogram was assessed by receiver operating characteristic (ROC) curve analysis, with calculation of the area under the curve (AUC). Calibration curves were generated to assess agreement between predicted probabilities and observed recurrence rates estimated by the Kaplan–Meier method, with patients grouped into deciles by predicted risk. Decision curve analysis (DCA) was performed to quantify the net clinical benefit across a range of threshold probabilities and to evaluate the practical utility of the model in clinical decision-making. Finally, the nomogram was graphically presented to facilitate individualized risk estimation.

## 3 Results

### 3.1 Clinicopathologic characteristics

**Figure 1:**
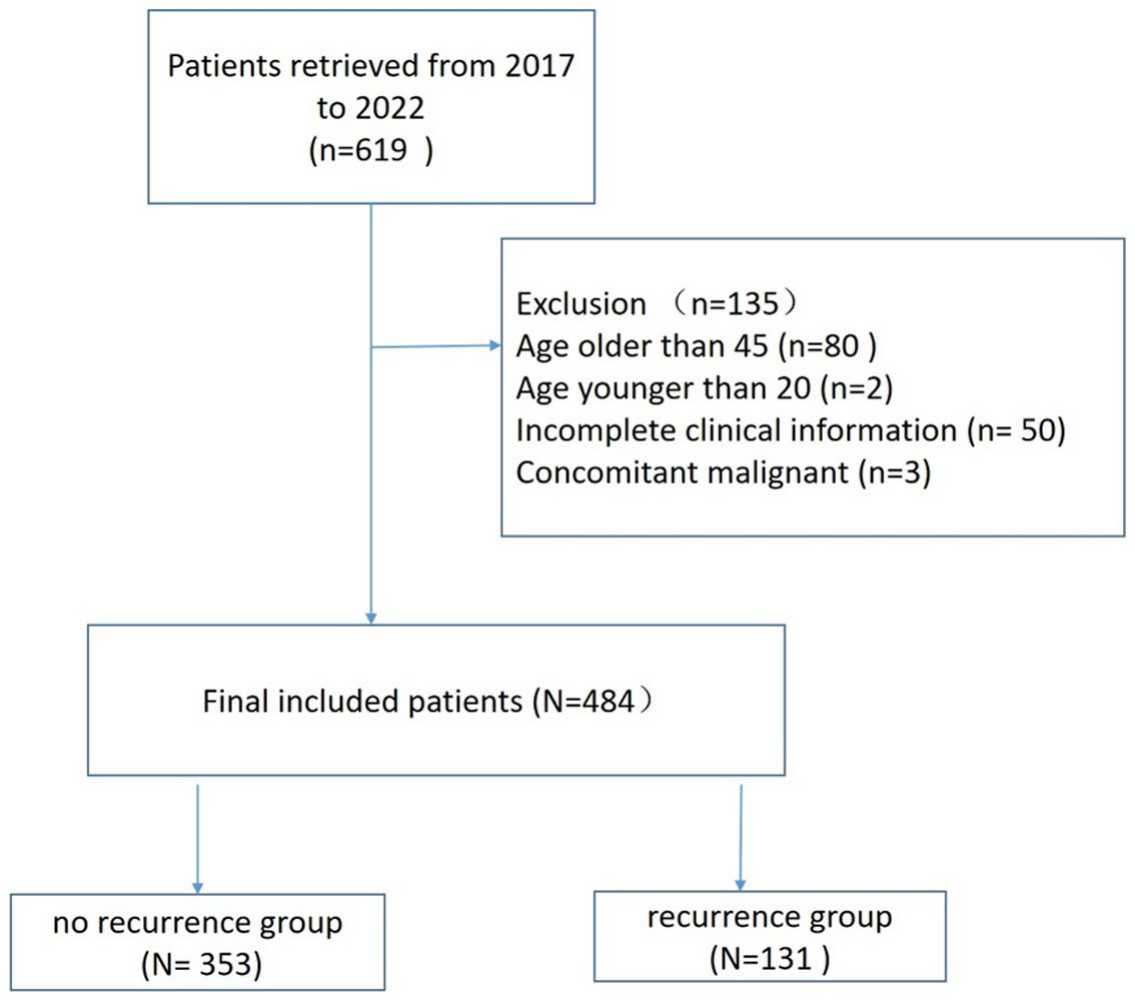
Flow diagram of patient selection and study inclusion.

A total of 619 patients who underwent laparoscopic adenomyomectomy with histopathological confirmation were initially identified. Of these, 82 patients were excluded due to age outside the eligibility range, 3 due to concomitant malignancy, and 50 due to incomplete clinical data. Ultimately, 484 patients were included in the final analysis, comprising 131 patients with symptomatic recurrence and 353 without recurrence (Figure 1).

Baseline clinicopathologic characteristics are summarized in Table 1. No significant differences were observed between the recurrence and nonrecurrence groups in age, body mass index (BMI), uterine volume, delivery mode, adenomyosis type, or lesion location. However, preoperative CA125 levels were significantly higher in the recurrence group (*p* < 0.001). Patients with recurrence were more likely to have a prior history of ovarian endometrioma (OMA) surgery (16.8% *vs*. 7.4%, *p* = 0.004) and concomitant OMA (38.9% vs. 21.8%, *p* < 0.001). In contrast, a substantially greater proportion of patients in the nonrecurrence group received postoperative hormonal suppression (83% *vs*. 54.2%, *p* < 0.001), and the duration of postoperative medical therapy was significantly longer in this group.

**Table 1:** Baseline characteristic comparison between the recurrence and the no recurrence group.

Variable	No recurrence group (*n* = 353)	Recurrence group (*n* = 131)	*p*-value
Age (year)	39.77 ± 3.94	39.33 ± (4.16)	0.280
BMI (kg/m^2^)	22.98 ± (2.95)	23.09 ± (2.80)	0.722
Uterus volume (cm^3^)	173.67 ± 82.86	180.68 ± (87.53)	0.416
Pre CA125 (U/L)	61.9 (35.0–114.0)	82.4 (48.2–151.0)	<0.001
Pre Hb (g/L)	117.58 ± (14.10)	117.31 ± (12.33)	0.848
Pre VAS	6.59 ± (2.46)	6.78 ± (2.26)	0.449
pre MVJ	4.81 ± (1.08)	4.93 ± (0.96)	0.262
Nullipara (*n*, %)	55 (15.6)	24 (18.3)	0.558
Multipara (*n*, %)	298 (84.4)	107 (81.7)	
Delivery mode (*n*, %)			0.775
None	57 (16.3)	24 (18.9)	
Cesarean section	169 (48.4)	58 (45.7)	
Vaginal delivery	123 (35.2)	45 (35.4)	
Abdominal surgery (%)			0.820
No	146 (41.4)	52 (39.7)	
Yes	207 (58.6)	79 (60.3)	
History of OMA surgery (%)			0.004
No	327 (92.6)	109 (83.2)	
Yes	26 (7.4)	22 (16.8)	
Previous medical treatment failure, *n* (%)	80 (22.7)	16 (12.2)	0.015
Type of adenomyosis (*n*, %)			0.921
Focal	239 (67.7)	90 (68.7)	
Diffuse	114 (32.3)	41 (31.3)	
Lesion_location (%)			0.078
Anterior wall	67 (19.0)	13 (9.9)	
Posterior wall	209 (59.2)	92 (70.2)	
Fundus	31 (8.8)	10 (7.6)	
Multiple locations	46 (13.0)	16 (12.2)	
Operation time (min)	156.91 ± 51.85	162.23 ± 60.06	0.338
Concomitant_OMA (*n*, %)			
No	276 (78.2)	80 (61.1)	<0.001
Yes	77 (21.8)	51 (38.9)	
Concomitant leiomyoma (*n*, %)			0.561
No	264 (74.8)	102 (77.9)	
Yes	89 (25.2)	29 (22.1)	
Concomitant DE (%)			0.858
No	282 (79.9)	103 (78.6)	
Yes	71 (20.1)	28 (21.4)	
Postoperative medication modality (*n*, %)			<0.001
Surgery only	60 (17.0)	60 (45.8)	
Combined with LNG-IUS	214 (60.6)	25 (19.1)	
Other hormone therapy	79 (22.4)	46 (35.1)	
Duration of postoperative therapy (*n*, %)			<0.001
None	84 (23.8)	73 (55.7)	
≤6 months	41 (11.6)	34 (26.0)	
6–12 months	56 (15.9)	13 (9.9)	
>12 months	172 (48.7)	11 (8.4)	
Follow-up time (month) median(range)	39.0 (36.0–45.0)	38.0 (37.0–40.5)	0.633

BMI Body mass index (calculated as weight in kilograms divided by the square of height in meters), CA125, Carbohydrate antigen 125; Hb, hemoglobin; VAS, Visual analog scale; MVJ, Mansfeld-Voda-Jorgensen Menstrual Bleeding Scale; OMA, ovarian endometrioma; DE, deep endometriosis; LNG-IUS Levonorgestrel-releasing intrauterine device.

The median follow-up time of the whole study was 39 months, and there was no significant difference in the follow-up time for women with and without recurrence (38.0 vs. 39.0 months, *p* = 0.633).

### 3.2 Univariate and multivariable cox regression

CA125 = 62.1 U/mL yielded the maximum Youden index of 0.166, corresponding to a sensitivity of 66.4% and a specificity of 50.1%, with an area under the curve (AUC) of 0.609 (95% CI: 0.554–0.663). Accordingly, preoperative CA125 was dichotomized at this threshold (>62.1 U/mL vs. ≤62.1 U/mL) for subsequent multivariable analysis and nomogram construction (Supplementary Figure S1).

Univariate Cox regression identified the following variables as potential predictors of symptomatic recurrence (*p* < 0.1): preoperative CA125 (>62.1 U/mL), previous surgical history of OMA, lesion location, concomitant OMA, postoperative medication modality, and duration of postoperative therapy (Table 2).

**Table 2:** Univariate and multivariate analyses of adenomyosis recurrence risk.

Variable	Univariate analysis		Multivariate analysis	
	HR (95% CI)	*p*-value	HR (95% CI)	*p*-value
Age	0.98 (0.94–1.02)	0.249		
BMI	1.01 (0.95–1.07)	0.787		
Multipara	0.89 (0.57–1.39)	0.618		
Delivery mode				
None	Reference			
Cesarean section	0.87 (0.54–1.40)	0.558		
Vaginal delivery	0.95 (0.58–1.57)	0.853		
Abdominal surgery	1.03 (0.72–1.46)	0.877		
Previous surgical history of OMA	1.98 (1.26–3.14)	0.003	1.89 (1.19–3)	0.007
Previous medical treatment failure	0.53 (0.31–0.89)	0.017		
Lesion location				
Anterior	Reference			
Posterior	2.00 (1.12–3.57)	0.019		
Fundus	1.44 (0.63–3.28)	0.388		
Multiple locations	1.65 (0.80–3.44)	0.178		
Type of adenomyosis				
Focal	Reference			
Diffuse	0.94 (0.65–1.36)	0.744		
Lesion size (cm)	0.94 (0.82–1.08)	0.392		
Preoperative uterine volume (cm^3^)	1.00 (1.00–1.00)	0.467		
Pre CA125 (U/ml)				
≤62.1	Reference			
>62.1	1.77 (1.23–2.55)	0.002	1.88 (1.3–2.71)	<0.001
Pre VAS score	1.03 (0.96–1.11)	0.410		
Pre MVJ score	1.08 (0.92–1.27)	0.367		
Concomitant OMA	1.90 (1.34–2.70)	<0.001	2.06 (1.42–3)	<0.001
Concomitant leiomyoma	0.84 (0.55–1.26)	0.394		
Concomitant DE	1.07 (0.71–1.63)	0.743		
Postoperative medication modality				
Surgery only	Reference		Reference	
Combined with LNG-IUS	0.17 (0.10–0.27)	<0.001	0.57 (0.31–1.05)	0.072
Other hormone therapy	0.69 (0.47–1.01)	0.054	0.93 (0.43–1.78)	0.712
Duration of postoperative therapy				
None	Reference		Reference	
≤6 months	0.98 (0.65–1.47)	0.922	0.79 (0.38–1.63)	0.522
6–12 month	0.32 (0.18–0.59)	<0.001	0.29 (0.13–0.64)	0.002
>12 month	0.10 (0.05–0.19)	<0.001	0.12 (0.06–0.28)	<0.001

HR, hazard ratio; CI, confidence interval.

Multivariate Cox regression further demonstrated that previous surgical history of OMA, preoperative CA125 level, concomitant OMA, and medication duration of postoperative therapy were independent predictors of symptomatic recurrence (*p* < 0.05) (Table 2).

### 3.3 Collinearity and interaction analysis

Collinearity assessment revealed that postoperative treatment (GVIF^(1/(2 × Df))^ = 1.485) and duration of therapy (GVIF^(1/(2 × Df))^ = 1.304) showed moderate inflation, consistent with their expected clinical correlation. All other predictors had GVIF^(1/(2 × Df))^ values below 1.10, indicating no meaningful collinearity (Supplementary Table S1).

Interaction analysis identified two significant pairwise interactions: treatment type × drug duration (LRT chi-square = 12.309, *p* < 0.001) and OMA surgery history × concomitant OMA (LRT chi-square = 7.692, *p* = 0.005) (Supplementary Table S2).

### 3.4 Nomogram development

**Figure 2:**
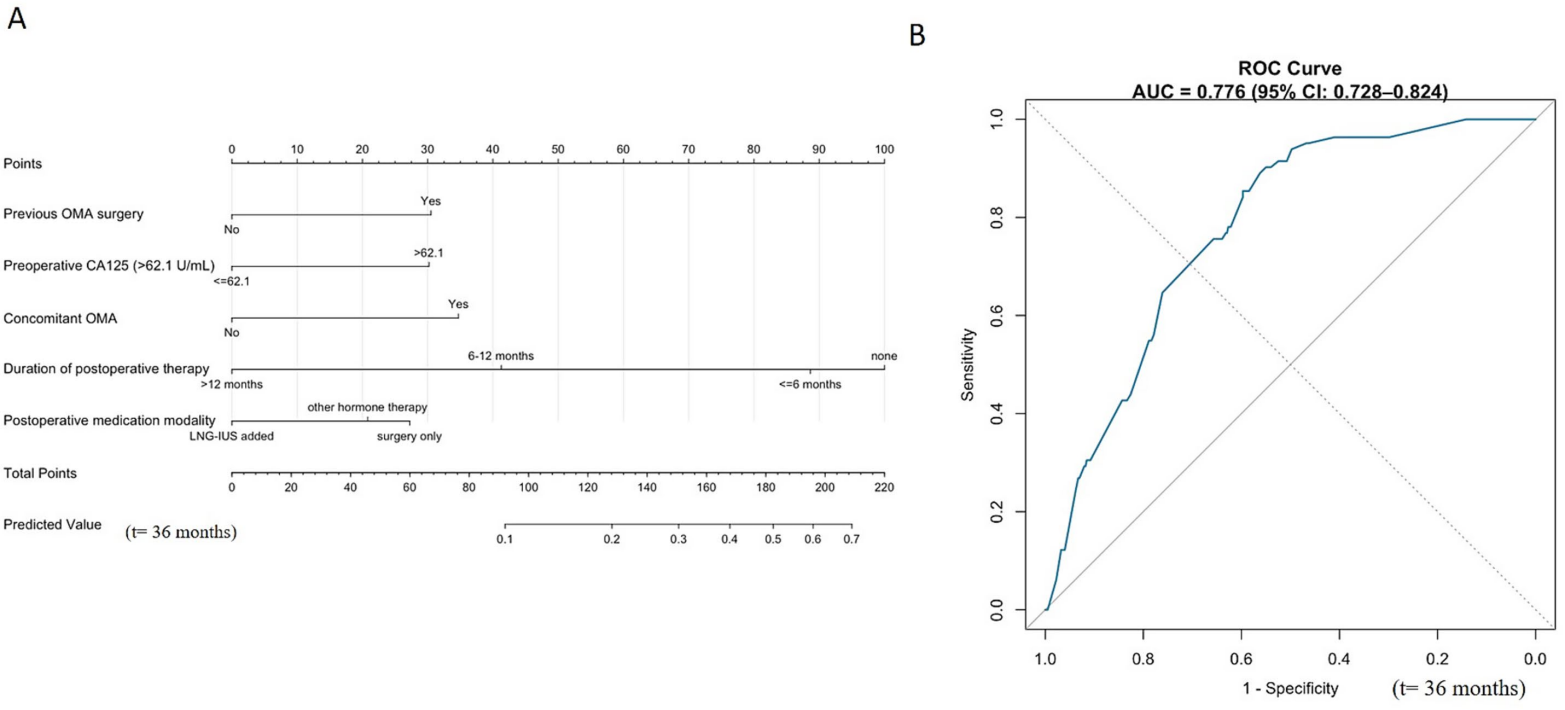
Nomogram for predicting symptomatic recurrence after laparoscopic adenomyomectomy **(A)**. Each predictor corresponds to a point value on the upper scale. The total score, obtained by summing individual points, is projected onto the lower probability scale to estimate the risk of postoperative symptomatic recurrence. Receiver operating characteristic (ROC) curves for (blue line) are shown **(B)**.

A nomogram was constructed to provide individualized symptomatic recurrence probability estimates. The nomogram incorporates five variables: previous OMA surgery, preoperative CA125 (>62.1 U/mL), concomitant OMA, duration of postoperative therapy, and postoperative medication modality. Each variable contributes a point score, with the sum of points mapping to a predicted recurrence probability ranging from approximately 0.1 to 0.7 (Figure 2A).

### 3.5 Model discrimination

The final model demonstrated good discriminative performance. The apparent C-index on the full sample was 0.772. Internal validation using 5-fold cross-validation yielded a mean C-index of 0.761 ± 0.038 (95% interval: 0.681–0.828), closely approximating the apparent C-index and indicating minimal overfitting. The distribution of C-index values across 25 folds was concentrated between 0.70 and 0.82, with no extreme outliers, confirming the stability of the model (Supplementary Figure S2).

The AUC was 0.776 (95% CI: 0.728–0.824), consistent with the overall C-index and indicating good discriminative ability at the clinically relevant prediction horizon (Figure 2B).

### 3.6 Model calibration

**Figure 3:**
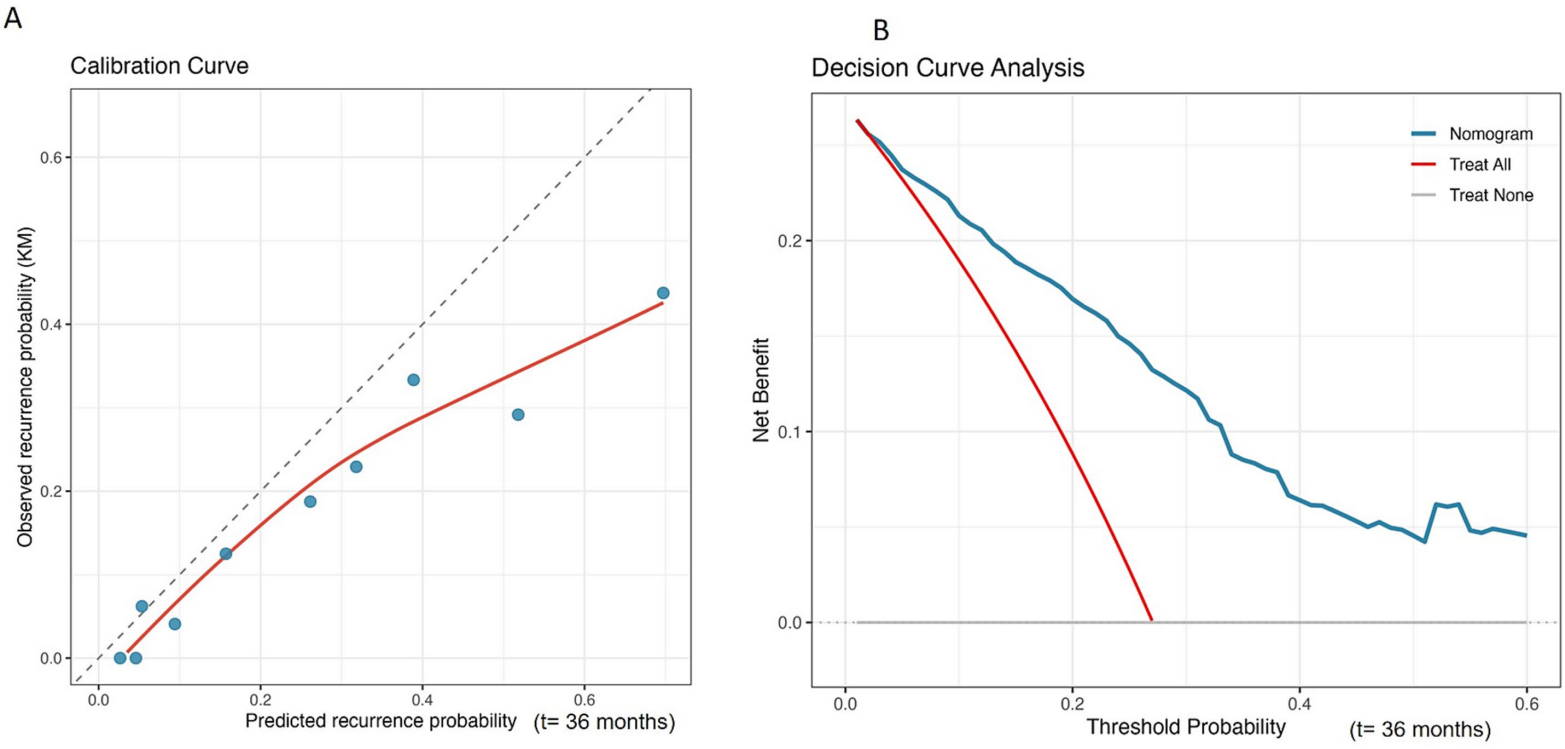
Calibration curve assessing agreement between predicted and observed recurrence probabilities **(A)**. Decision curve analysis (DCA) evaluating the clinical utility of the nomogram **(B)**.

The calibration curve assessed agreement between predicted recurrence probabilities and observed rates estimated by the Kaplan–Meier method, with patients grouped into deciles by predicted risk. In the low-risk region (predicted probability 0–0.15), the model demonstrated good calibration, with predicted and observed probabilities closely aligned along the diagonal. In the intermediate-to-high risk region (predicted probability 0.20–0.45), the LOESS-smoothed calibration curve fell consistently below the 45° reference line, indicating a tendency toward overprediction (Figure 3A). In the highest-risk group (predicted probability ~0.65–0.70), calibration was again reasonable. Overall, the model’s calibration performance was acceptable in the low-risk range but exhibited moderate overprediction at intermediate risk levels, a pattern likely attributable to the apparent (non-bootstrap-corrected) nature of the calibration assessment.

### 3.7 Model clinical utility

Decision curve analysis demonstrated that the nomogram provided positive net benefit across threshold probabilities from approximately 0.01 to 0.60, consistently outperforming both the “treat all” and “treat none” strategies. The net benefit advantage was most pronounced in the threshold range of 0.05–0.30, representing the range most clinically relevant for treatment decision-making (Figure 3B).

### 3.8 Sensitivity analysis

To assess robustness to residual confounding, an inverse probability of treatment weighting (IPTW) sensitivity analysis was performed using 15 variables not included in the final model as confounders. After applying IPTW, the standardized mean differences of most residual confounders were reduced below the 0.10 threshold, indicating satisfactory balance. The comparison of hazard ratios between the main analysis and the IPTW-weighted analysis is shown in Supplementary Table S3.

Three predictors demonstrated consistent findings across both analyses. Preoperative CA125 > 62.1 U/mL maintained a nearly identical HR (main: 1.75, IPTW: 1.88, both *p* < 0.01). Concomitant OMA remained significant in both models, though the HR attenuated from 2.06 to 1.53 after weighting, suggesting partial confounding by residual factors. Drug durations of 6–12 months (main: HR 0.29, IPTW: HR 0.25) and >12 months (main: HR 0.12, IPTW: HR 0.10) remianed consistent and highly significant in both analyses.

## 4 Discussion

Because conservative surgery for adenomyosis cannot completely eradicate all ectopic endometrial tissue, even in focal disease, postoperative recurrence remains a substantial challenge. Reported recurrence rates are approximately 9% following complete excision and 19% after partial excision (16). Accurate delineation of lesion borders and separation of infiltrated myometrium from normal tissue are particularly difficult in diffuse adenomyosis, contributing to residual disease and recurrence.

To our knowledge, this is the first study to develop and validate a nomogram for predicting symptomatic recurrence after fertility-preserving surgery for adenomyosis. We identified five independent predictors: previous surgical history of OMA, preoperative CA125, concomitant OMA, postoperative medication modality, and duration of postoperative therapy. The nomogram assigns factor weights objectively via Cox regression coefficients to achieve standardized, repeatable risk stratification. Many conventional clinical scoring systems adopt arbitrary equal weights without statistical support. In contrast, our nomogram weights predictors based on actual recurrence hazard ratios, yielding more precise risk quantification.

Adenomyosis and endometriosis share similar pathogenic mechanisms and frequently coexist, with evidence suggesting bidirectional influence (17). Prior studies have shown that adenomyosis independently increases the risk of endometriosis recurrence after surgery during long-term follow-up (18). Conversely, concomitant endometriosis has been reported as an independent risk factor for relapse after laparoscopic adenomyomectomy (19). Consistent with these findings, our results demonstrated that both prior OMA surgery and concomitant OMA significantly increased the risk of postoperative recurrence (aHR: 1.89, 95% CI: 1.19–3, *p* = 0.007; and aHR: 2.06, 95% CI: 1.42–3, *p* < 0.001, respectively), in agreement with Zhu et al. (20). Even if the effect of this variable remains statistically significant after weighted correction, it is still necessary to objectively acknowledge the interference of confounding factors on the size of the predictor’s effect: combined OMA remains an independent risk factor for postoperative recurrence, but the actual clinical effect intensity is weaker than the results of the unweighted original model. Based on this, when using this nomogram to assess the individual recurrence risk, the confounding interference caused by the imbalance of baseline clinical characteristics should be fully considered, and the predictive weight of ovarian endometriotic cysts should not be overly magnified.

Serum CA125 is widely used to differentiate adenomyosis from uterine fibroids (21). Elevated CA125 levels correlate with dysmenorrhea severity and disease extent (22), and have been associated with recurrence after adenomyomectomy (19) as well as therapeutic response (23). It has been validated that the commonly used preoperative CA125 threshold was sensitive to assess the risk of incidence and timing of symptom recurrence of adenomyosis (24). The optimal cutoff identified in our study was 62.1 U/mL (sensitivity 66.4%, specificity 50.1%). In our multivariate analysis, elevated preoperative CA125 (>62.1 U/mL) independently predicted recurrence (aHR: 1.88, 95% CI: 1.3–2.71, *p* = 0.001). Incorporation of CA125 into the nomogram enhances individualized risk estimation, and continued postoperative monitoring may facilitate early detection of recurrence.

Adjuvant hormonal therapy plays a critical role in recurrence prevention. Initiating medical treatment immediately after surgery has been associated with lower recurrence rates (25), although optimal regimens remain debated. A previous study demonstrated that GnRH-a combined with LNG-IUS achieved significantly lower recurrence rates than GnRH-a alone (26), suggesting that sustained hormonal suppression improves long-term symptom control. Post-adenomyomectomy uterine size may be more favorable for LNG-IUS placement, reducing expulsion risk (27). LNG-IUS provides continuous local progestogenic suppression of residual or *de novo* lesions (27), making it a potentially superior postoperative option. In our study, postoperative medication modality (surgery only vs. other hormone therapy vs. LNG-IUS) was not independently significant in the multivariable model, though it was retained in nomogram given its clinical importance and significant interaction with drug duration. When applying this model in clinical practice, the individual scores of treatment types or medication duration cannot be interpreted in isolation. Instead, both should be combined to comprehensively assess the risk of recurrence, thereby compensating for the deviation in effect interpretation caused by not including interaction terms in the model.

Increasing evidence supports long-term adjuvant therapy to minimize recurrence after laparoscopic adenomyomectomy (28). Prolonged progestin therapy has been shown to provide sustained symptom relief in adenomyosis (29). Consistent with this, our findings demonstrated that longer postoperative medication duration, especially > 12 months, significantly reduced recurrence risk (aHR: 0.12, 95% CI: 0.06–0.28, *p* < 0.001). The nomogram highlights duration of adjuvant therapy as a key determinant of durable symptom control (30).

This study has limitations. One limitation is that imaging confirmation was not used to evaluate patients at every visit. Thus, our assessment of the recurrence of adenomyoma was not clinically definitive. Additionally, the assessment of dysmenorrhea was subjective, so differences in the experience of patients may have affected the results. Secondly, its retrospective single-center design introduces potential selection, recall, and follow-up biases. Thirdly, the sample size, although substantial, may limit statistical power. And direct pairwise comparison between the nomogram, clinician prediction and simplified scoring was not conducted, which constitutes a key limitation of the current study. Lastly, there is no multicenter external data validation and prospective study, which may also limit its applicability. Therefore, our findings require validation in large-scale, multicenter, prospective studies with longer follow-up before their definitive clinical utility can be confirmed. Despite the above limitations, this nomogram acts as an objective auxiliary tool rather than a replacement for clinical experience. It may offer tentative reference information to support clinicians when contemplating personalized postoperative management strategies aimed at mitigating long-term recurrence risk among patients at elevated risk.

## 5 Conclusion

We developed and validated a nomogram incorporating five independent predictors, including previous surgical history of ovarian endometrioma, preoperative CA125, concomitant ovarian endometrioma, postoperative medication modality, and duration of postoperative therapy, to estimate the risk of symptomatic recurrence after laparoscopic adenomyomectomy, which required multicenter external validation to confirm its clinical utility.

## Data Availability

The raw data supporting the conclusions of this article will be made available by the authors, without undue reservation.
