# Supplementary material for: Development and validation of a nomogram to predict symptomatic recurrence following laparoscopic adenomyomectomy
Source: Front Med (Lausanne). 2026 Jul 16;13:1869800. doi: 10.3389/fmed.2026.1869800 (PMC13422411; doi:10.3389/fmed.2026.1869800)
Supplement: Supplementary file 1 [file Supplementary_file_1.docx]

**Supplementary Table S1. Generalized Variance Inflation Factors (GVIF) for Multivariable Model Predictors**

| **Variable** | **GVIF** | **Df** | **GVIF^(1/(2×Df))** |
| --- | --- | --- | --- |
| Previous OMA surgery | 1.076 | 1 | 1.037 |
| Prior drug failure | 1.071 | 1 | 1.035 |
| Lesion location | 1.145 | 3 | 1.023 |
| Preoperative CA125 | 1.061 | 1 | 1.030 |
| Concomitant OMA | 1.202 | 1 | 1.096 |
| Postoperative treatment | 4.858 | 2 | 1.485 |
| Duration of postoperative therapy | 4.909 | 3 | 1.304 |

GVIF: generalized variance inflation factor; Df: degrees of freedom. Threshold for concern: GVIF^(1/(2×Df)) > √5 ≈ 2.24.

**Supplementary Table S2. Interaction Analysis Results (Likelihood Ratio Test)**

| **Interaction** | **LRT Chi-square** | **df** | **P value** | **Conclusion** |
| --- | --- | --- | --- | --- |
| Treatment × Drug duration | 12.309 | 1 | <0.001 | Significant interaction |
| OMA surgery × Concomitant OMA | 7.692 | 1 | 0.005 | Significant interaction |

LRT: likelihood ratio test.

**Supplementary Table S3. Comparison of Hazard Ratios: Main Analysis vs. IPTW Sensitivity Analysis**

| **Variable** | **Main Analysis HR (95% CI)** | **P** | **IPTW-Weighted HR (95% CI)** | **P** |
| --- | --- | --- | --- | --- |
| Previous OMA surgery | 2.09 (1.30–3.35) | 0.002 | 1.44 (0.99–2.09) | 0.059 |
| Preoperative CA125 >62.1 U/mL | 2.06 (1.27–3.36) | 0.004 | 2.03 (1.32–3.14) | 0.001 |
| Concomitant OMA | 2.08 (1.43–3.03) | <0.001 | 1.54 (1.14–2.09) | 0.005 |
| Drug duration: ≤6 months | 0.83 (0.40–1.70) | 0.605 | 0.70 (0.36–1.35) | 0.287 |
| Drug duration: 6–12 months | 0.29 (0.13–0.63) | 0.002 | 0.25 (0.12–0.52) | <0.001 |
| Drug duration: >12 months | 0.12 (0.06–0.28) | <0.001 | 0.10 (0.05–0.23) | <0.001 |
| Treatment: other hormone therapy | 0.93 (0.45–1.89) | 0.835 | 1.33 (0.69–2.56) | 0.395 |
| Treatment: LNG-IUS added | 0.61 (0.33–1.13) | 0.114 | 1.02 (0.54–1.92) | 0.961 |

IPTW: inverse probability of treatment weighting. Bold values indicate p<0.05.

**Supplementary Figure S1**


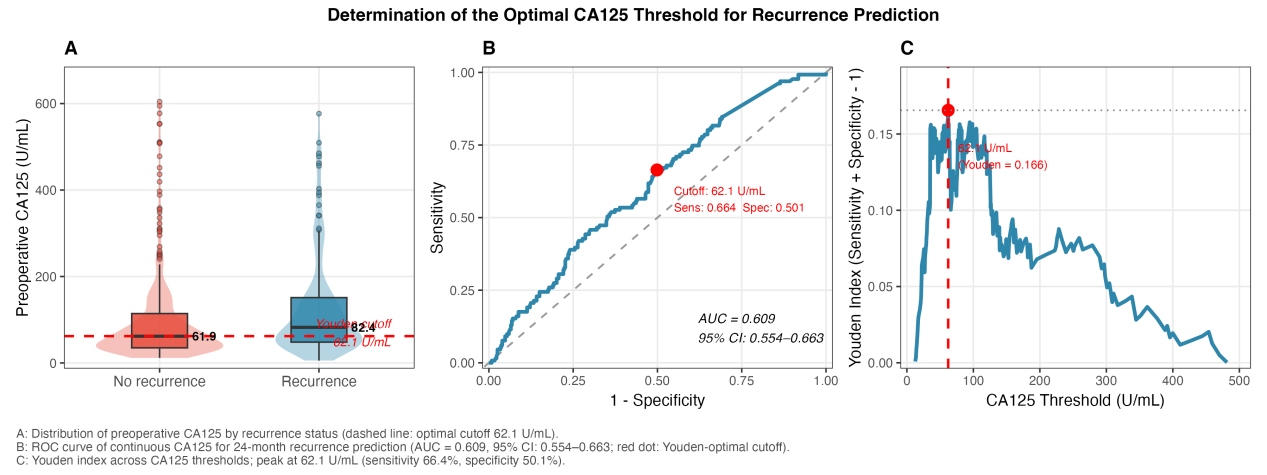


**Supplementary Figure S2 CV C-index**

**
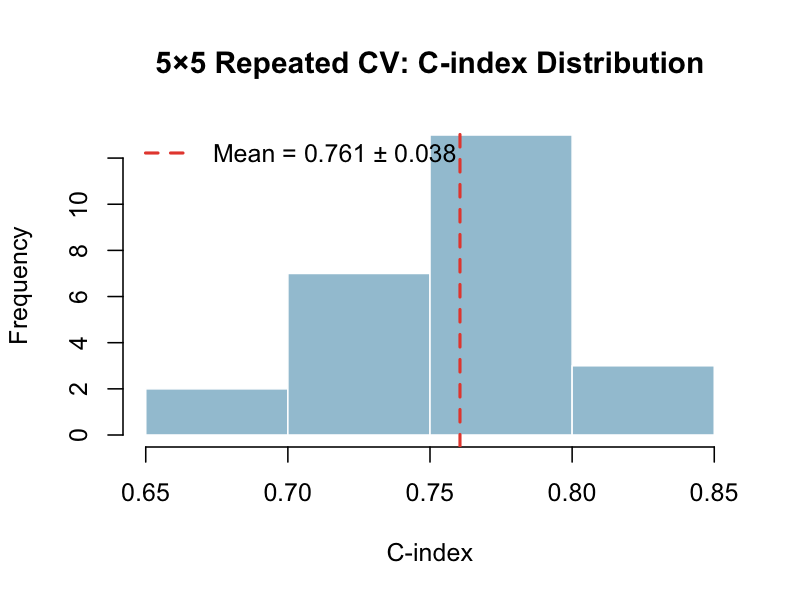
**
